# Neural Adjoint Inverse Design of Three‐Dimensional Nanophotonic Devices

**DOI:** 10.1002/nap2.70334

**Published:** 2026-10-16

**Authors:** Chanik Kang, Hyewon Suk, Ikbeom Jang, Haejun Chung

**Affiliations:** ^1^ Department of Artificial Intelligence Hanyang University Seoul Korea; ^2^ Department of Artificial Intelligence Semiconductor Engineering Hanyang University Seoul Korea; ^3^ Department of Computer Engineering Hankuk University of Foreign Studies Yongin Korea; ^4^ Department of Electronic Engineering Hanyang University Seoul Korea

**Keywords:** adjoint method, fourier neural operator, inverse design, meta‐optics, scientific machine learning, surrogate modeling

## Abstract

Volumetric meta‐optics have emerged as a compact high‐performance platform for advanced imaging, color routing in image sensors, and on‐chip wavefront control. Realizing such devices depends on solving a high‐dimensional nonconvex inverse‐design problem governed by Maxwell's equations. Adjoint optimization is the standard route to gradients, yet broadband three‐dimensional (3D) design typically requires thousands of full‐wave forward/adjoint simulations, making industrial‐scale optimization prohibitively slow. To address this bottleneck, a neural adjoint method is introduced, in which a solver‐supervised Fourier neural operator (FNO) directly predicts dense, per‐voxel 3D adjoint gradient fields from a voxelized permittivity volume. To preserve the sharp, resonance‐driven sensitivity peaks that single‐pass operator networks tend to oversmooth, a stage‐wise FNO is proposed, in which successive stages refine residual errors with progressively higher‐frequency emphasis. A domain‐specific dataset is curated from paired forward/adjoint finite‐difference time‐domain (FDTD) simulations spanning three volumetric tasks: spectral sorting (color routers), achromatic focusing (metalenses), and waveguide mode conversion. Across all benchmarks, the surrogate‐driven optimization attains figures of merit within 86%–99% of rigorous FDTD‐based adjoint optimization whilst reducing end‐to‐end design time from hours to seconds, with speedups exceeding $10^{4}\times$. These results establish a pathway toward fast large‐scale volumetric meta‐optical design enabled by neural‐operator surrogates.

## Graphical abstract

A stage‐wise Fourier neural operator is trained on FDTD adjoint gradients of volumetric meta‐optics, each stage refining residuals under progressively enlarged Fourier‐mode cutoffs. Given any permittivity volume, the model reconstructs the 3D gradient field, from coarse envelope to resonance peaks, within a second, driving inverse design to 86–99% of FDTD‐optimized performance, 10,000‐fold faster.

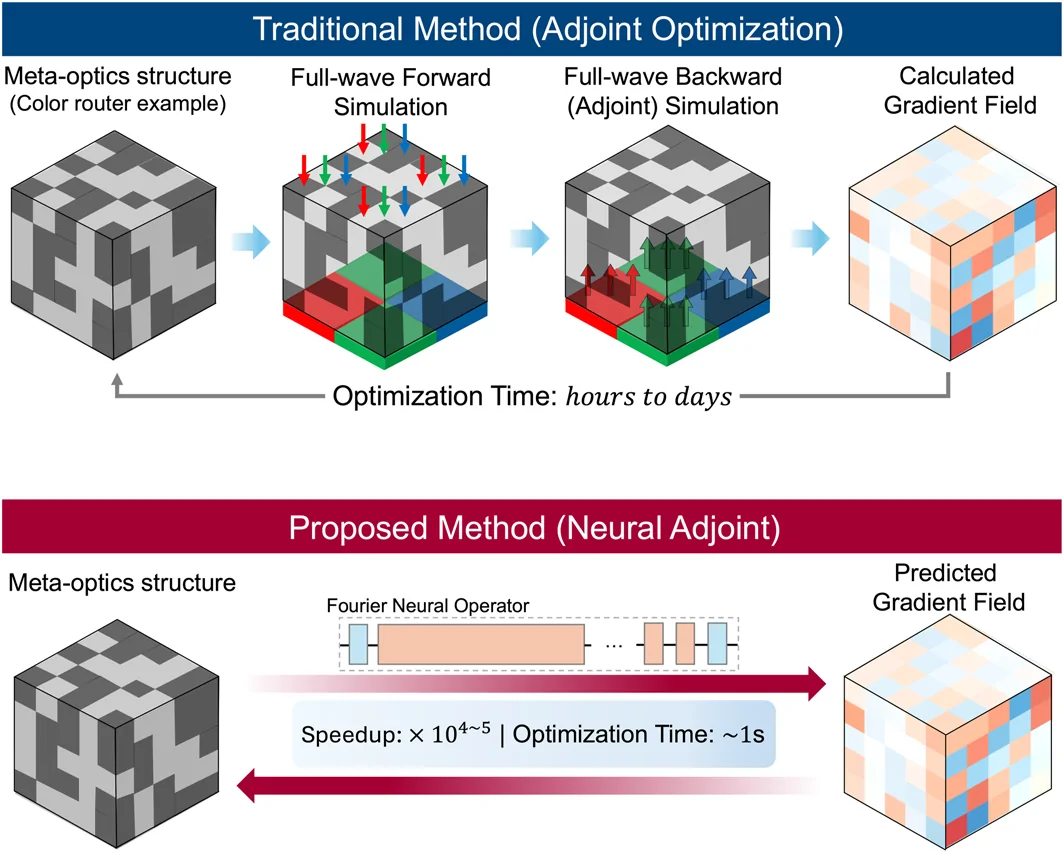

## 1 Introduction

Optical‐system miniaturization has accelerated the commercialization of meta‐optics, marking a major transition from academic research to industrial‐scale manufacturing [1, 2, 3, 4]. In particular, the display and semiconductor industries are increasingly adopting 3D volumetric meta‐optics, with color routers for complementary metal‐oxide‐semiconductor (CMOS) image sensors being a prominent example, to address the fundamental efficiency constraints of conventional dye‐based color filters [5, 6, 7]. Unlike thin diffractive components, volumetric meta‐optical devices leverage complex multiple scattering within micrometer‐scale volumes to enable precise control of wavefronts and spectra [8, 9, 10]. For deployment in consumer electronics, these devices must meet stringent performance requirements across broad wavelength ranges, necessitating an unprecedented degree of precision in the design and optimization process [11].

However, industrial deployment of these devices remains limited by substantial computational overhead. The inverse design of 3D volumetric meta‐optics is typically a high‐dimensional nonconvex optimization problem constrained by Maxwell's equations [12]. The adjoint method is the standard approach for computing gradients, requiring two full‐wave simulations per iteration (one forward and one adjoint) [13]. Although adjoint optimization is computationally efficient in terms of gradient evaluation, the overall cost of rigorous 3D solvers such as finite‐difference time‐domain (FDTD) grows rapidly with problem size [14]. In addition, commercial designs must be optimized over the full visible band, which typically requires separate simulations for each wavelength or color channel [15]. As a result, a single optimization run can involve tens of thousands of full‐wave simulations and take days to weeks to complete. This turnaround time poses a major obstacle for high‐throughput industrial research and development, where rapid prototyping and iterative refinement are critical.

To mitigate this cost, deep learning has emerged as a promising alternative. Generative models (GANs [16, 17, 18], diffusion [19, 20, 21, 22]) offer rapid one‐shot designs, but they suffer from a precision‐speed trade‐off [23]. Conversely, numerical optimization guarantees physical optimality but is constrained by the extreme slowness of the solver [24].

To resolve this precision‐speed trade‐off, we propose the neural adjoint method, a surrogate framework that retains the precision of iterative gradient‐based optimization whilst drastically reducing its solver cost. Our approach retains the conventional gradient‐based optimization loop and replaces only the per‐iteration gradient evaluation, namely the paired forward/adjoint full‐wave solves, with a Fourier neural operator (FNO)‐based surrogate [25, 26]. In contrast to end‐to‐end generative approaches, which substitute the entire iterative design pipeline with a learned generator, this decouples the per‐iteration cost of optimization from the cost of full‐wave simulation. The 3D volumetric gradient field is predicted within a second, supporting a solver‐reduced optimization loop that drastically shortens the design turnaround time.

The proposed framework addresses the challenges of volumetric inverse design through three key contributions. First, inverse design is formulated around dense 3D adjoint sensitivities, and a neural operator is trained to predict full volumetric adjoint gradient fields directly from voxelized devices, enabling a single surrogate architecture, trained separately for each task, to serve heterogeneous meta‐optical objectives. Second, to counteract the low‐frequency bias of single‐pass operator networks, a stage‐wise Fourier neural operator (SW‐FNO) is introduced, in which successive stages refine residual errors with progressively higher‐frequency emphasis, thereby improving the recovery of localized high‐magnitude sensitivity peaks that are critical for topology refinement. Third, the inferred gradients are integrated into a gradient‐based optimization loop, enabling rapid design iteration without per‐iteration full‐wave Maxwell solves. The framework is validated on three representative 3D inverse‐design tasks: RGB color routing [7, 27, 28], achromatic metalens design [29, 30, 31], and waveguide mode conversion [32, 33, 34, 35]. Across all tasks, the neural adjoint method achieves performance comparable to rigorous FDTD‐based adjoint optimization whilst reducing end‐to‐end design time by several orders of magnitude, with speedups of up to $∼10^{4}$–$10^{5}\times$. By mitigating the bottleneck of repeated full‐wave simulations, these results suggest a practical pathway toward rapid large‐scale 3D meta‐optical design enabled by AI‐accelerated scientific computing.

The present work builds on several lines of inquiry. Adjoint‐based optimization is widely used for large‐scale photonic and meta‐optical inverse design, enabling efficient gradient computation with only two full‐wave simulations per objective evaluation [13, 36, 37, 38, 39, 40, 41]; however, the overall runtime remains dominated by repeated full‐wave simulations across many iterations and wavelengths, motivating efforts to reduce the cost of solver‐in‐the‐loop optimization. Neural operators, including FNO, have emerged as mesh‐agnostic surrogates for PDE‐governed systems, such as electromagnetics [42, 43], with most prior work focused on predicting fields or spectra to reduce the cost of repeated simulations [12, 44, 45, 46, 47, 48]; more recently, gradient learning has been investigated as an alternative, in which adjoint sensitivities required by gradient‐based optimizers are directly regressed [49, 50, 51, 52]. A complementary line of work introduces fabrication awareness directly into the objective itself through density filtering, projection schemes, length‐scale control, and three‐field robust formulations [53, 54, 55, 56]; because these formulations modify the objective rather than the gradient evaluation routine, they compose orthogonally with the present approach, which focuses on accelerating the gradient evaluation itself and leaves the choice of objective formulation to the user.

Building on gradient learning with neural operators, the present work focuses on 3D volumetric inverse design across diverse physics‐based objectives, including focusing, spectral routing, and mode conversion. Extending neural adjoint surrogates from low‐dimensional or 2D problems to 3D introduces practical challenges related to representational capacity, preservation of high‐spatial‐frequency sensitivity, and scalability. These challenges are addressed through a volumetric multiobjective surrogate formulation that predicts 3D gradient fields on voxel grids and generalizes across multiple classes of objectives, a stage‐wise refinement scheme with progressive frequency emphasis that better preserves sharp sensitivity peaks critical for faster convergence, and a controllable synthetic data‐generation pipeline that produces diverse, smoothly varying topologies together with solver‐generated gradients for supervised training.

## 2 Background and Problem Formulation

This section formulates the volumetric inverse design problem as a constrained optimization task governed by Maxwell's equations, outlines the dominant computational cost, and contrasts the present work with prior efforts on adjoint optimization and neural surrogates.

### 2.1 Inverse Design for Diverse Physical Objectives

Inverse design in meta‐optics and nanophotonics seeks a device parameterization (e.g., a 2D/3D permittivity distribution) that maximizes or minimizes a physics‐based objective function [14]. A common approach is gradient‐based adjoint optimization: given an objective functional J(x) evaluated by solving Maxwell's equations, the adjoint method computes $\nabla_{x}J$ using only two full‐wave simulations, a forward solve to obtain the electromagnetic fields and an adjoint solve to back‐propagate sensitivity, enabling scalable updates even when x is high‐dimensional [13]. In this work, this conventional formulation is used as the reference pipeline, and the focus is on accelerating the expensive field/gradient evaluations.

As illustrated in Figure 1, volumetric inverse design is considered within a three‐dimensional physical domain $\Omega \;\subset \;R^{3}$. The design variable is the spatial permittivity distribution ϵ(r), which is discretized as a voxel grid $x\in R^{N}$ with $N=N_{x}N_{y}N_{z}$ degrees of freedom. For a given design x and wavelength λ, a full‐wave Maxwell solver computes the complex electric field E(r;x,λ), from which task‐specific figures of merit (FoMs) are defined.

**FIGURE 1:**
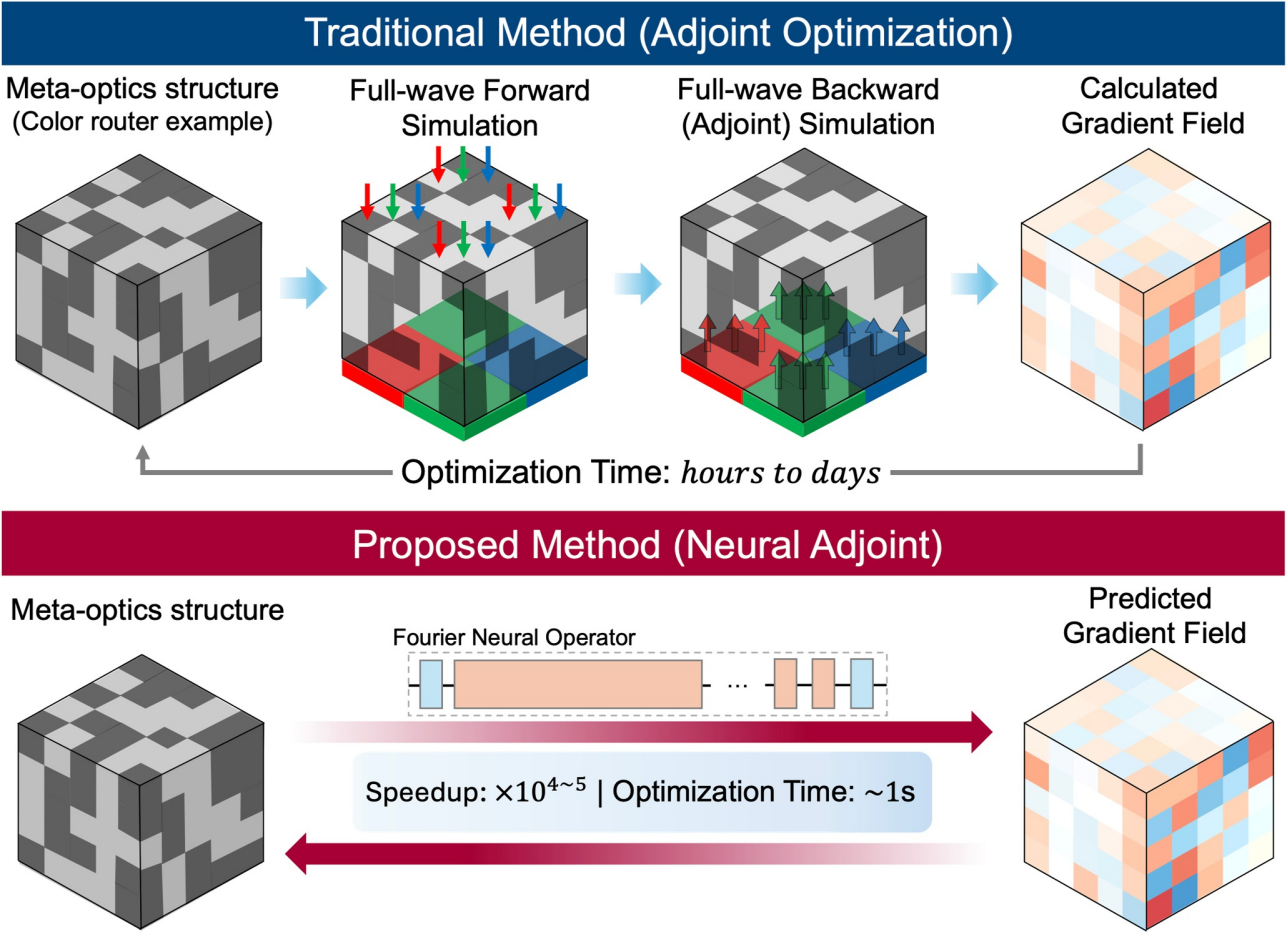
Breaking the simulation bottleneck in 3D meta‐optics. (Top) Conventional adjoint optimization relies on iterative full‐wave high‐fidelity simulations (Maxwell solvers) to compute gradients, requiring hours to days per design. (Bottom) The proposed neural adjoint method replaces the solver with a Fourier neural operator, inferring volumetric gradient fields in seconds.

Three representative classes of meta‐optics/nanophotonics objectives are studied as visualized in Figure 2. For the metalens task, the field intensity within a prescribed focal volume $V_{f}\;\subset \;\Omega$ is maximized across K target wavelengths ${\left\{\lambda_{k}\right\}}_{k=1}^{K}$:

(1) $$J_{\text{focus}}\left(x\right)=\sum_{k=1}^{K}\int_{V_{f}}{\left\|E\left(r;x,\lambda_{k}\right)\right\|}^{2}\;dr.$$

**FIGURE 2:**
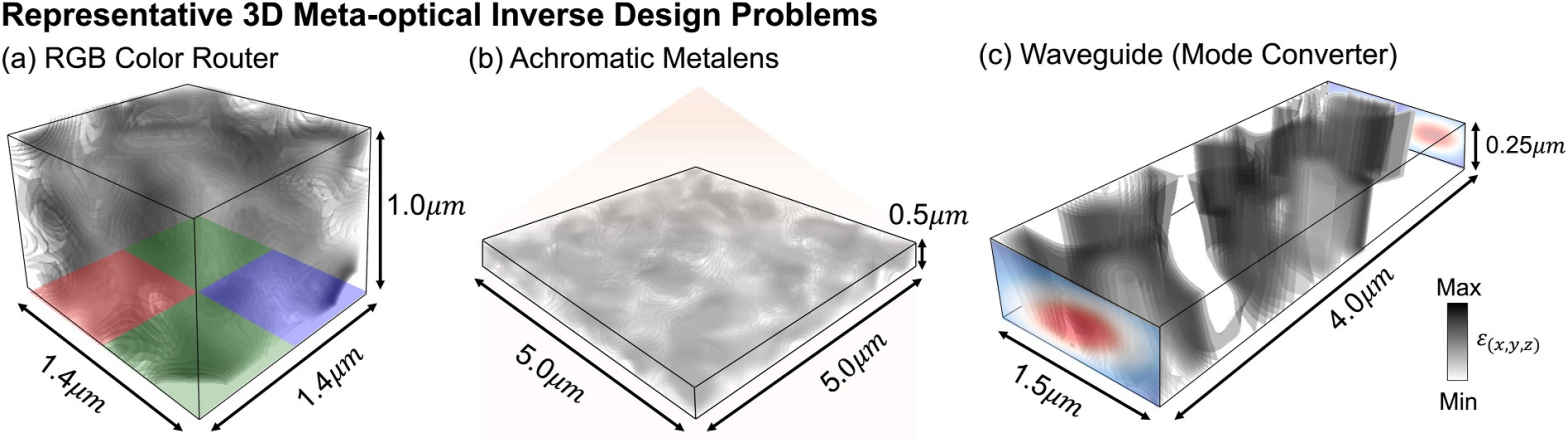
3D meta‐optical inverse‐design tasks considered in this work: (a) spectral sorting (color router), (b) light focusing (metalens), and (c) guided‐wave mode conversion (waveguide mode converter).

For the color router task, K target wavelengths ${\left\{\lambda_{k}\right\}}_{k=1}^{K}$ are spatially sorted and the field intensity integrated over wavelength‐specific target regions ${\left\{R_{k}\right\}}_{k=1}^{K}$ is maximized:

(2) $$J_{\text{sort}}\left(x\right)=\sum_{k=1}^{K}\int_{R_{k}}{\left\|E\left(r;x,\lambda_{k}\right)\right\|}^{2}\;dr.$$

For the waveguide mode converter, the modal overlap between the output field on the cross‐section $S_{\text{out}}$ and a target mode profile $\Psi_{\text{tgt}}$ is maximized:

(3) $$J_{\text{mode}}\left(x\right)={\left|\int_{S_{\text{out}}}E\left(r;x,\lambda \right)\;\cdot \;\Psi_{\text{tgt}}^{*}\left(r\right)\;dr\right|}^{2}.$$

Although the objectives differ across tasks, they are all governed by the same frequency‐domain Maxwell equation:

(4) $$\nabla \times \nabla \times E\left(r\right)-k_{0}^{2}\epsilon \left(r\right)E\left(r\right)=-i\omega \mu_{0}J_{\text{src}}\left(r\right),$$

where $k_{0}=2\pi /\lambda$.

### 2.2 The Adjoint‐Solver Bottleneck

To optimize the objectives above, gradient‐based inverse design typically employs the adjoint method (also known as the adjoint‐variable method) to compute the sensitivity of the FoM with respect to the design parameters [13, 57]. Let $g=\nabla_{x}J$ denote the gradient. In electromagnetic adjoint formulations, g can be expressed as a point‐wise dot product between the forward field $E_{\text{fwd}}$ and the adjoint field $E_{\text{adj}}$:

(5) $$g\left(r\right)\;\propto \;Re\left[E_{\text{fwd}}\left(r\right)\;\cdot \;E_{\text{adj}}\left(r\right)\right],$$

where the adjoint source term is determined by the particular FoM.

Importantly, independent of the chosen FoM, the dominant cost in adjoint‐based optimization arises from repeatedly solving the full‐wave Maxwell system in Equation (4) to obtain the forward and adjoint fields. In 3D, high‐resolution, and broadband settings, these repeated full‐wave computations become the primary bottleneck, rendering conventional iterative optimization prohibitively expensive in practice.

### 2.3 Neural Surrogate for Adjoint Gradients

The objective is to approximate the adjoint‐to‐gradient mapping $G:x\;\mapsto \;\nabla_{x}J$ using a neural surrogate $f_{\theta }$. Although the scalar FoM J differs across applications, the volumetric gradient field $g=\nabla_{x}J$ encodes the local sensitivity information required by gradient‐based optimizers. Accordingly, the problem is formulated as dense field prediction:

(6) $$\hat{g}=f_{\theta }\left(x\right)\approx \nabla_{x}J\in R^{N_{x}\times N_{y}\times N_{z}}.$$

Each task uses a single task‐specific surrogate, and wavelength is not an explicit network input. Instead, wavelength information enters through the design objective. For the multi‐wavelength FoMs of Equations (1) and (2), the supervision label is the gradient of the aggregated objective, $g=\nabla_{x}J=\sum_{k=1}^{K}\nabla_{x}J_{k}$, where $J_{k}$ denotes the single‐wavelength summand at $\lambda_{k}$. By linearity of differentiation, this aggregation is exact and coincides with the field required by the design‐update rule. In the remainder of this paper, $f_{\theta }$ is treated as a drop‐in replacement for repeated adjoint solves within iterative optimization. The model architecture and training procedure are described in the Methodology section.

## 3 Methodology

This section presents the neural adjoint method for accelerating volumetric meta‐optical inverse design. As illustrated in Figure 3, a stage‐wise Fourier neural operator (SW‐FNO) is introduced that predicts 3D adjoint gradient fields via coarse‐to‐fine residual refinement, improving the recovery of localized high‐sensitivity structures. A solver‐reduced optimization loop is then described that updates the design using the inferred gradients, enabling rapid iterative refinement without repeatedly invoking full‐wave forward/adjoint simulations.

**FIGURE 3:**
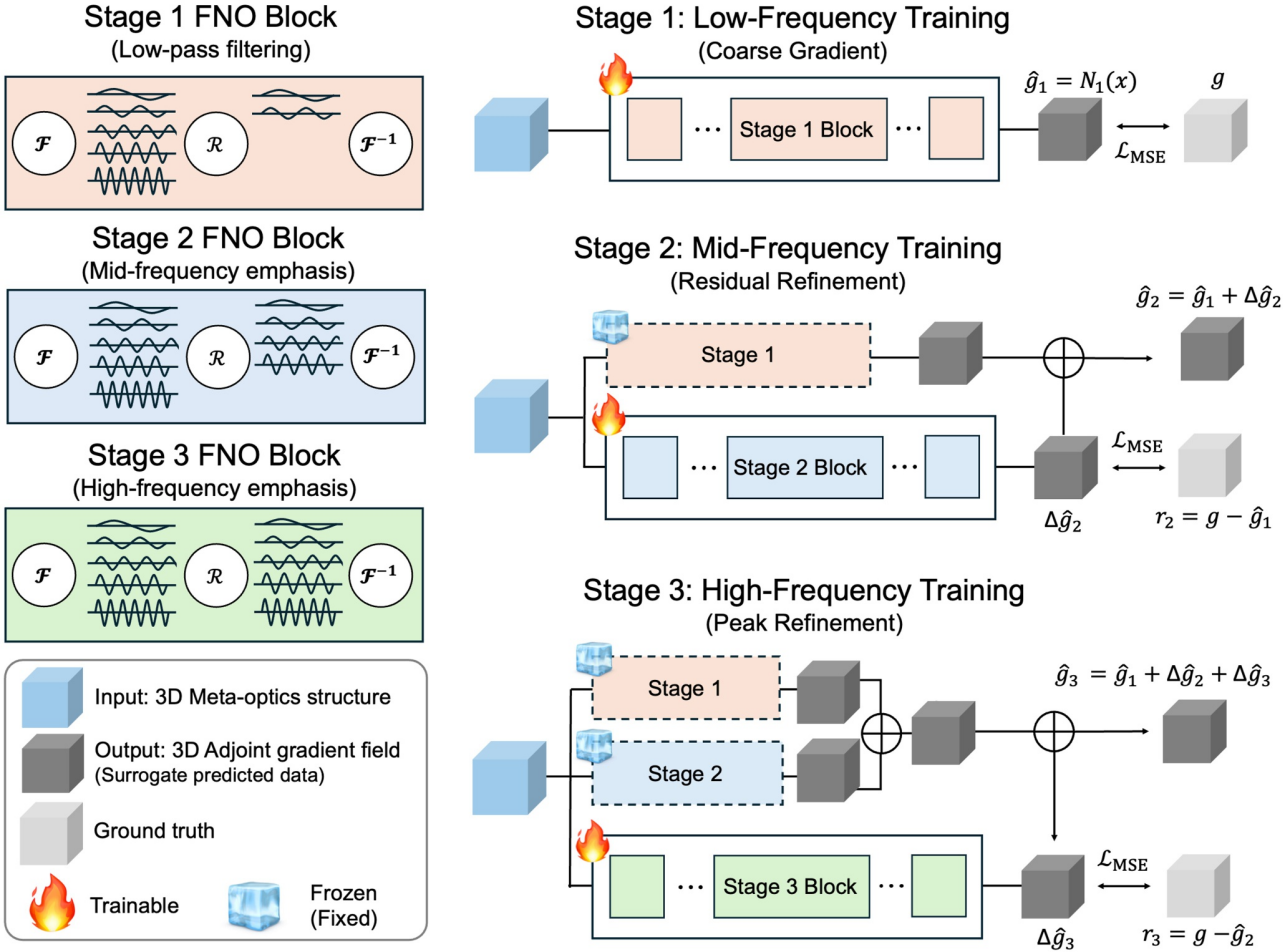
Stage‐wise Fourier neural operator (SW‐FNO). Overview of the stage‐wise training scheme for predicting dense 3D adjoint gradient fields from a voxelized meta‐optics structure. Each stage employs an FNO block operating in the Fourier domain, with frequency emphasis progressively shifted from low to mid to high bands (left: simplified nested‐cutoff FNO blocks). Training proceeds sequentially: Stage 1 learns a coarse low‐frequency prediction; subsequent stages are trained on residual targets to refine missing components, whereas earlier stages are frozen. The final prediction is obtained by summing the stage outputs, enabling accurate reconstruction across the full frequency spectrum whilst preserving sharp high‐frequency sensitivity peaks. Each stage retains all Fourier modes up to its per‐stage cutoff; the low/mid/high labels denote each stage's effective frequency emphasis induced by residual training.

### 3.1 Stage‐Wise Fourier Neural Operator (SW‐FNO)

To map a voxelized permittivity distribution $x\in R^{N_{x}\times N_{y}\times N_{z}}$ to a dense adjoint gradient field g, the surrogate must represent electromagnetic phenomena, including wave propagation, scattering, and resonance, with the latter producing sharp high‐spatial‐frequency sensitivity peaks. In practice, a single‐stage FNO often under‐represents these high‐frequency components, resulting in over‐smoothed gradient fields and, consequently, degraded optimization performance [58].

To address this limitation, a stage‐wise refinement strategy is adopted that better matches the multiscale nature of 3D volumetric scattering [59]. The proposed SW‐FNO performs progressive refinement: it first captures coarse, global trends, and then successively adds residual corrections to recover fine‐scale high‐spatial‐frequency components [60]. Formally,

(7) $$\hat{g}={\hat{g}}_{1}+\sum_{s=2}^{S}\Delta {\hat{g}}_{s},\;{\hat{g}}_{1}=N_{1}\left(x\right),\;\Delta {\hat{g}}_{s}=N_{s}\left(x\right)\approx r_{s},$$

where S is the number of stages and $\Delta {\hat{g}}_{s}$ denotes the residual correction predicted at stage s. This additive coarse‐to‐fine decomposition encourages later stages to focus on what earlier stages miss, enabling sharper recovery of high‐frequency gradient features whilst preserving global consistency of the predicted field. The three stages use nested, progressively enlarged Fourier‐mode cutoffs rather than architecturally disjoint band‐pass filters. The low/mid/high terminology refers to each stage's effective emphasis, which is induced by residual training. Because the frozen earlier stages already capture the content representable within their smaller cutoffs, the learning signal of each later stage concentrates on the modes newly admitted between the previous and the current cutoffs, and each stage's correction is concentrated in, though not strictly limited to, this newly admitted range. The enlarged cutoff also retains the lower modes, which allows later stages to refine residual low‐frequency errors left by earlier stages. The cutoffs follow a simple rule anchored to the single‐stage baseline. The mid‐stage cutoff equals the truncation cutoff of the single‐stage FNO baseline. The Stage 1 cutoff retains roughly 60%–78% of the baseline cutoff per axis, so the first stage models only the coarse global envelope. The Stage 3 cutoff extends 20%–33% beyond the baseline, approaching the largest mode count representable on the voxel grid. For the color router, for example, the cutoffs are refs [6, 8, 10] and refs. [10, 12] modes per axis, and the per‐task values are listed in Supporting Information S1: Table S1.

#### 3.1.1 Stage 1: Global Physics Learning

The first stage uses a 3D FNO to capture the dominant low‐spatial‐frequency global structure of the adjoint gradient field. These components reflect long‐range interactions, wave propagation effects, and coarse sensitivity patterns throughout the volume. For the l‐th Fourier layer, the feature update is defined as

(8) $$v_{l+1}\left(r\right)=\sigma \left(W_{l}\;v_{l}\left(r\right)+F^{-1}\left(R_{l}\left(k\right)⊙F\left(v_{l}\right)\left(k\right)\right)\left(r\right)\right),$$

where F and $F^{-1}$ denote the fast Fourier transform (FFT) and inverse FFT, $W_{l}$ is a pointwise linear projection, and $R_{l}$ is a complex‐valued spectral weight tensor defined on the truncated set of Fourier modes (up to $k_{\text{base}}$). Operating in the Fourier domain yields a global convolution that captures nonlocal physics‐driven correlations.

The Stage 1 predictor produces a coarse global estimate of the gradient field,

(9) $${\hat{g}}_{1}=N_{1}\left(x\right),$$

which provides a globally consistent initialization for subsequent residual stages and stabilizes training by avoiding the need to learn fine‐scale corrections from scratch. With a limited mode budget $\left(k_{\text{base}}\right)$ and the spectral bias of operator networks, ${\hat{g}}_{1}$ may inevitably smooth out boundary‐aligned variations and localized resonant features that require higher spectral resolution.

#### 3.1.2 Stage 2: Mid‐Frequency Residual Refinement

Although Stage 1 captures dominant global sensitivity patterns, its prediction ${\hat{g}}_{1}$ can under‐represent mid‐frequency structures around material interfaces, boundaries, and transition regions due to low‐mode truncation and spectral bias. Therefore, Stage 2 predicts a residual correction on top of the frozen Stage 1 estimate, following the residual‐learning principle [61]. The Stage 1 residual is defined as $r_{2}=g-{\hat{g}}_{1}$, where g denotes the ground‐truth adjoint gradient field obtained from the FDTD solver. Stage 2 then learns this residual via

(10) $$\Delta {\hat{g}}_{2}=N_{2}\left(x\right),$$

and the refined estimate is obtained as

(11) $${\hat{g}}_{2}={\hat{g}}_{1}+\Delta {\hat{g}}_{2}.$$

Because the frozen Stage 1 already captures the content representable within its smaller cutoff, the learning signal of $N_{2}$ concentrates on the modes admitted between the Stage 1 and Stage 2 cutoffs (Supporting Information S1: Table S1).

#### 3.1.3 Stage 3: High‐Frequency Peak Refinement

Even after Stage 2 recovers boundary‐aligned mid‐frequency residuals, the target adjoint gradient field may still exhibit sharp highly localized sensitivity peaks at higher spatial frequencies. These peaks often arise from abrupt spatial variations (e.g., resonance‐induced confinement and interface discontinuities) and indicate fine‐scale regions where topology updates must remain responsive.

Standard FNOs exhibit a low‐frequency bias and, under finite mode truncation, tend to smooth spiky high‐frequency components, which can underestimate gradient magnitudes near localized maxima. To better preserve these high‐frequency extrema, Stage 3 predicts an additional residual correction on top of the frozen Stage 2 estimate. The Stage 2 residual is $r_{3}=g-{\hat{g}}_{2}$, and Stage 3 learns this residual as

(12) $$\Delta {\hat{g}}_{3}=N_{3}\left(x\right),$$

yielding the final prediction

(13) $${\hat{g}}_{\text{final}}={\hat{g}}_{2}+\Delta {\hat{g}}_{3}.$$

By explicitly modeling the remaining residual, Stage 3 improves the recovery of high‐magnitude localized peaks that are critical for fine‐scale topology refinement, helping to avoid attenuated sensitivities and to preserve reliable update directions. Similarly, the learning signal of $N_{3}$ concentrates on the modes admitted between the Stage 2 and Stage 3 cutoffs (Supporting Information S1: Table S1).

### 3.2 Learning Adjoint Gradients From Full‐Wave Simulations

The surrogate $f_{\theta }$ is trained on paired samples of voxelized device representations and their corresponding adjoint gradients, $\left\{\left(x_{i},g_{i}\right)\right\}$. Each $g_{i}$ is obtained from a conventional adjoint formulation using the open‐source FDTD package Meep [62]: for each design $x_{i}$, a forward simulation is run to evaluate the objective and an adjoint simulation is run to compute the volumetric sensitivity field. The learning problem is formulated as dense regression. Given x, the model predicts the full 3D gradient field $\hat{g}=f_{\theta }\left(x\right)$ and is optimized to match the solver‐generated g. Dataset sizes are task‐dependent, with 600 waveguide, 1100 metalens, and 3300 color‐router pairs used for training and validation. Each training set was grown until the validation gradient fidelity entered the high‐fidelity regime and further samples yielded diminishing gains (Supporting Information S1: Section 5.1, Table S3).

## 4 Results and Discussion

The neural adjoint method is empirically evaluated on three volumetric meta‐optical inverse‐design tasks. The experimental study is organized around three questions. The first concerns generalization: whether a single neural framework can be applied to physically distinct inverse‐design problems using a consistent architecture, without task‐specific modifications. The second concerns efficiency: how much speedup the proposed method provides relative to conventional FDTD‐based adjoint optimization. The third concerns component analysis: whether multistage refinement and high‐frequency boosting improve gradient fidelity and final design performance, and what is the trade‐off with model size and inference latency.

### 4.1 Baselines and Evaluation Metrics

The neural adjoint method is compared against four baselines covering both physics‐grounded optimization and learning‐based surrogates. The first is the adjoint method (FDTD reference), a conventional adjoint optimization pipeline that computes gradients via paired forward/adjoint full‐wave simulations and updates the design with a gradient‐based optimizer; this provides the physically accurate benchmark FoM for comparison. The surrogate‐driven pipeline shares this optimization loop, including the optimizer and all update settings, and differs only in the source of the gradient (Supporting Information S1: Section 6, Table S4). The second is the one‐stage FNO, a vanilla Fourier neural operator that predicts the full 3D adjoint gradient field in a single pass without stage‐wise refinement. The third is the triple‐stage FNO (SW‐FNO, the proposed model), which cascades three independent FNO blocks with progressively larger Fourier‐mode budgets to enable coarse‐to‐fine refinement from low‐to‐high‐frequency content. The fourth is a 3D U‐Net, a volumetric encoder–decoder architecture with skip connections trained to predict the dense adjoint gradient field. For evaluation, two complementary metrics are reported: functional fidelity, defined as the ratio of the final FoM achieved by the surrogate‐driven optimization to the FDTD benchmark; and structural fidelity, defined as the cosine similarity between the predicted and ground‐truth gradient fields (for gradient inference) or between the final voxelized designs (for end‐to‐end optimization).

### 4.2 Adjoint Gradient Inference Across Diverse Benchmarks

The proposed framework is evaluated on three representative high‐dimensional inverse‐design problems: an RGB color router, an achromatic metalens, and a waveguide mode converter. Both structural fidelity and functional performance are analyzed.

To quantify gradient fidelity, the cosine similarity between the surrogate‐predicted gradient fields and the rigorous FDTD adjoint gradients is computed, directly measuring the alignment of update directions used by gradient‐based optimizers. A global rescaling of the gradient is absorbed by the optimizer step size, whereas directional errors directly alter the update, so cosine similarity is the decisive measure for optimization performance. Magnitude fidelity is quantified separately in the Supporting Information. As summarized in Table 1, the proposed method consistently achieves the highest cosine similarity across all three tasks, outperforming both the standard one‐stage FNO and 3D U‐Net baselines. Qualitatively, Figure 4 supports this conclusion: baseline models tend to produce over‐smoothed gradient fields that attenuate localized extrema, whereas the proposed method reconstructs sharper peak structures and fine‐grained spatial features across all benchmarks.

**TABLE 1:** Comparison of gradient prediction performance (cosine similarity, %).

Model	Color router		Metalens		Waveguide	
	Mean (std)	Max	Mean (std)	Max	Mean (std)	Max
U‐net	61.4 (13.7)	86.4	53.6 (6.5)	69.1	32.6 (23.9)	71.9
FNO	66.3 (13.1)	89.7	73.5 (10.3)	90.0	46.4 (25.2)	80.5
Ours	82.3 (13.7)	99.8	87.6 (7.5)	98.2	87.9 (30.3)	99.4

**FIGURE 4:**
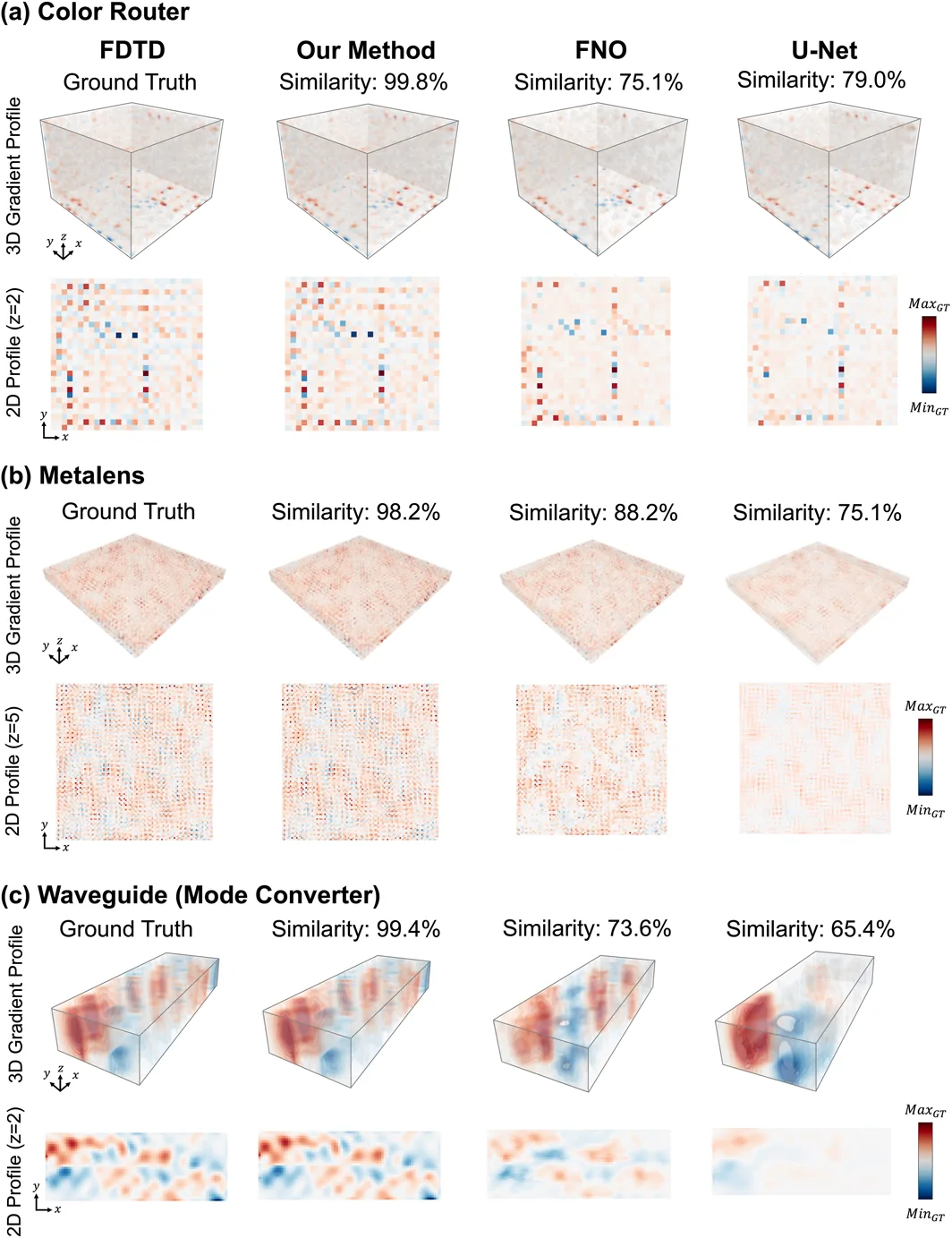
Qualitative comparison of volumetric adjoint‐gradient predictions. Ground‐truth gradient fields are visualized alongside predictions from the proposed method, a standard FNO, and a 3D U‐Net across three tasks: (a) RGB color router, (b) metalens, and (c) waveguide mode converter. Each panel displays a 3D volumetric rendering and a corresponding 2D cross‐sectional slice at the indicated z‐depth. Although baseline models (FNO and U‐Net) tend to produce over‐smoothed fields that miss critical high‐frequency components, the proposed method accurately reconstructs sharp gradient peaks and fine‐grained spatial features, achieving maximum cosine similarity scores exceeding 98% across all benchmarks.

### 4.3 Surrogate‐Driven Inverse Design

To assess end‐to‐end utility for inverse design, two complementary outcomes are reported: (i) the final design performance achieved by surrogate‐driven optimization and (ii) the total wall‐clock design time required to reach the final design. Performance is reported as relative optimality (FoM and structure similarity), defined as the ratio of the achieved FoM/structure similarity to the FDTD baseline. Results across tasks are summarized in Table 2, and a qualitative example of an optimized color router is shown in Figure 5.

**TABLE 2:** Relative optimality and design speed.

Task	Rel. opt. (%)		Design time		
	FoM	Struct.	FDTD	Ours	Speedup
Color router	95.9	72.1	3.4 h	1.0 s	×12,240
Metalens	86.0	83.6	40.1 h	1.8 s	×80,200
Waveguide	98.8	91.2	11.5 h	0.7 s	×59,143

*Note:* Surrogate‐driven designs are compared against the FDTD benchmark. All methods are run for a fixed number of optimization iterations: 50 (color router), 100 (metalens), and 30 (waveguide).

**FIGURE 5:**
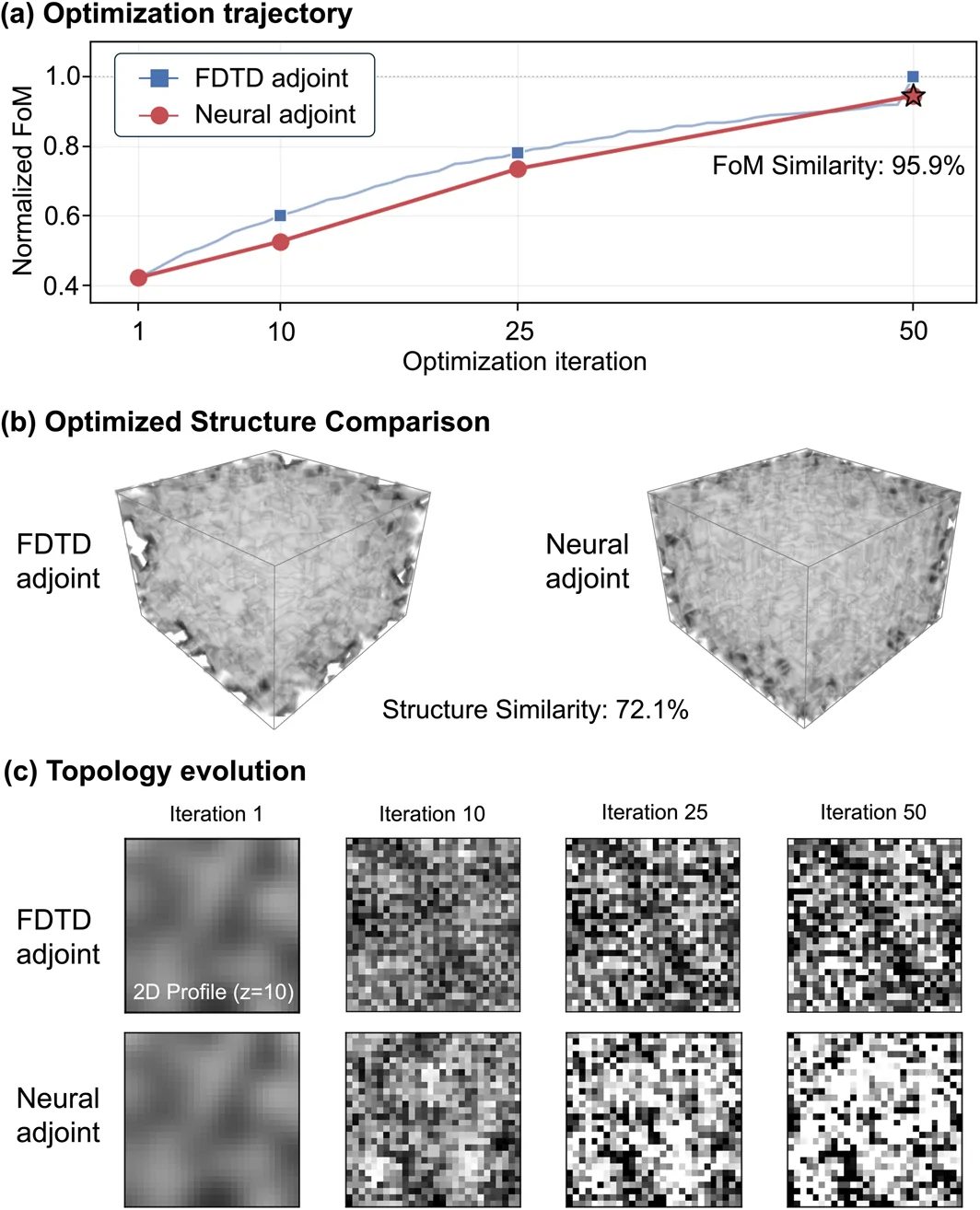
Surrogate‐driven inverse design on the RGB color router. (a) Normalized figure‐of‐merit (FoM) trajectory over 50 optimization iterations for FDTD‐based adjoint optimization (blue squares) and the proposed Neural Adjoint method (red circles), both initialized from an identical random permittivity distribution. The two pipelines follow closely matched convergence curves, and the neural adjoint final design attains 95.9% of the FDTD‐optimized FoM. (b) Volumetric renderings of the final optimized permittivity distributions. Although the resulting geometries are not identical (structure similarity 72.1%), they achieve comparable functional performance, consistent with the known nonuniqueness of photonic inverse‐design solutions. (c) Topology evolution shown as *z* = 10 cross‐sectional slices at iterations 1, 10, 25, and 50 for both methods. The two trajectories exhibit similar coarse‐to‐fine refinement patterns, indicating that the surrogate‐driven update directions traverse a comparable optimization landscape to the rigorous FDTD reference, whilst reducing per‐iteration wall‐clock cost by roughly four orders of magnitude.

Across all three benchmarks, the proposed method achieves high relative optimality whilst substantially reducing design time. The resulting designs remain close to the FDTD‐optimized solutions in terms of final performance, despite eliminating repeated full‐wave forward/adjoint solves during optimization. This indicates that the neural adjoint surrogate supports a shared optimization pipeline that transfers across disparate meta‐optical objectives. Notably, the color‐router and waveguide tasks reach ≳95% relative FoM, whilst structural similarity remains lower, consistent with the known nonuniqueness of inverse‐designed photonic solutions: different topologies can satisfy the same physical objective. A spectral‐coverage analysis of the surrogate‐driven trajectory further shows that the iterates remain within the training envelope through the structure‐forming phase and progressively exceed it as binarization proceeds, whereas the closely matched trajectories of Figure 5a confirm that this late‐phase mismatch does not degrade optimization fidelity (Supporting Information S1: Section 7).

### 4.4 Computational Efficiency

The primary advantage of the proposed method is the elimination of the simulation bottleneck. Compared to conventional solver‐in‐the‐loop adjoint optimization, the surrogate yields orders‐of‐magnitude speedups on all tasks (Table 2 and Figure 6), reducing design time from hours to seconds. Although a single iteration of 3D FDTD simulation for the color router takes approximately minutes to hours on a workstation (CPU: AMD Ryzen Threadripper PRO 9995WX), neural adjoint inference takes only milliseconds on a single GPU (NVIDIA H200).

**FIGURE 6:**
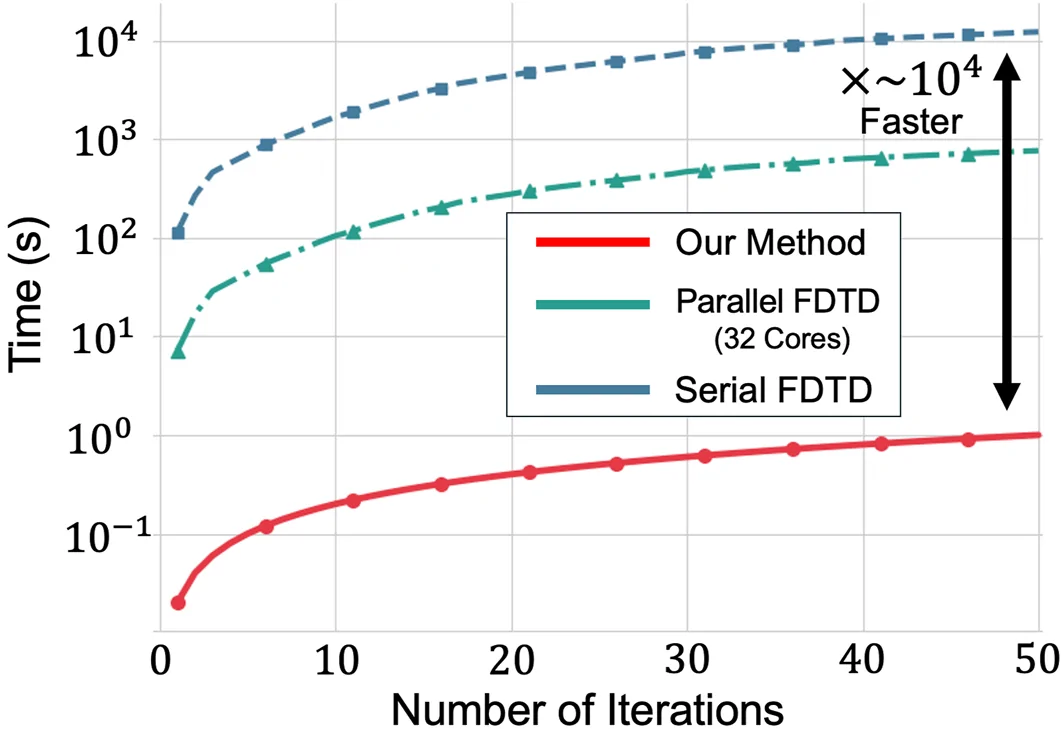
Optimization time comparison. Wall‐clock time versus the number of optimization iterations for the neural adjoint method and FDTD‐based adjoint optimization (serial and 32‐core parallel implementations). Across iterations, the proposed method remains orders of magnitude faster, achieving up to $∼10^{4}\times$ speedup over serial FDTD.

### 4.5 Effect of Multi‐Stage Refinement

Table 3 shows that the substantial gain emerges at three stages, with two‐stage refinement (L →H) alone yielding no improvement over the single‐stage baseline. This nonmonotonic pattern indicates that direct low‐to‐high residual learning is insufficient: the wide spectral gap between coarse global features and sharp resonance‐driven peaks requires an intermediate mid‐frequency stage that bridges the two scales. With a three‐stage cascade (L → M → H), the model recovers localized high‐magnitude extrema that the single‐pass and two‐stage variants attenuate, consistent with the multiscale nature of 3D volumetric scattering. This behavior is also consistent with the spectral‐bias and frequency‐principle literature on neural networks [58, 63]. The rule used to select the per‐stage cutoffs is stated in Section 3.1 and verified per task in Supporting Information S1: Section 2.1.

**TABLE 3:** Ablation of the number of refinement stages in SW‐FNO (1–4) on the metalens task.

#Stages	Stage emphasis	#Params (*M*) ↓	Latency (ms) ↓	Rel. opt. FoM (%) ↑
1	L	7.09	4.8	73.5
2	L → H	14.71	7.8	73.1
3 (Ours)	L → M → H	21.80	10.9	87.6
4	L → LM → HM → H	42.53	14.0	88.7

*Note:* The Fourier‐mode emphasis of each stage is denoted by L (low), M (mid), and H (high); intermediate emphasis levels are abbreviated as LM (low–mid) and HM (high–mid).

Notably, moving from three to four stages increases relative FoM by only 1.1 percentage points, whilst substantially increasing both the number of parameters and inference latency. The four‐stage variant also covers the same spectral range with different interior cutoffs, and its similar relative FoM (88.7% vs. 87.6%) indicates that the final performance is not hyper‐sensitive to the exact placement of the interior cutoffs. Given this trade‐off, the three‐stage configuration is adopted as the default, providing a favorable balance between accuracy and computational overhead.

### 4.6 Manufacturability Considerations and Outlook

The designs in this work are freeform volumetric structures, and fabrication‐constrained design is beyond the scope of this proof‐of‐concept study. Freeform inverse‐designed structures have been used as unconstrained performance references because their large design freedom reduces the geometric bias introduced by predefined or fabrication‐constrained parameterizations [64, 65]. Such optimized structures are not rigorous upper bounds by themselves. Their performance can instead be interpreted through comparison with independently derived physical bounds [66, 67], and bound‐guided inverse design can explicitly target performance near such limits [65]. Accordingly, the present freeform designs should be interpreted as empirical performance benchmarks for a specified design volume, material system, and objective, against which future fabrication‐constrained implementations can be evaluated.

Fabrication routes for this class of meta‐optics are developing rapidly. Metasurface color routers have been fabricated and characterized [7, 10, 28]. Industrial‐scale manufacturing has been demonstrated using roll‐to‐roll [1] and wafer‐scale [2] processes. Two‐photon polymerization has been used to produce 3D‐patterned inverse‐designed meta‐optics [68] and 3D‐printed multilayer metalenses [31], and its capabilities for imaging optics have been reviewed in Ref. [69]. Layer‐by‐layer stacking also underlies the volumetric platforms demonstrated in Refs. [8, 39].

Relative to a reduced two‐dimensional model, which treats one direction as invariant, a three‐dimensional model can represent fabrication deviations throughout the volume, including interface displacements, thickness variations, layer misalignment, and shrinkage. Shrinkage and feature‐size mismatch have been identified as likely contributors to the reduced experimental contrast of 3D‐printed meta‐optics [68]. The effects of these deviations depend on the material distribution, operating wavelengths, and device function, so dimensionality alone does not determine whether a design is more or less tolerant.

Neural adjoint acceleration is also compatible in principle with fabrication‐constrained design. Training pairs could be generated from process‐relevant geometries, and constraints, such as prescribed layer thicknesses, material choices, and minimum feature sizes [55, 56], would be imposed through the design parameterization or update rule. The predicted gradients would then be propagated to the design variables by the chain rule as is already done for the threshold projection in the present pipeline (Supporting Information S1: Section 6).

Perturbation‐insensitive solutions can be targeted by jointly optimizing nominal, eroded, and dilated realizations, together with process‐specific perturbations, under an average or worst‐case objective [53, 54, 55]. Each scenario requires its own gradient evaluation, so such formulations multiply the per‐iteration cost of FDTD‐based adjoint optimization, which already requires 3.4–40.1 h per design in the present benchmarks (Table 2). A surrogate trained and validated on the perturbed geometries could perform these evaluations in milliseconds, and the gradient fidelity reported in Section 4.2 is a prerequisite for this acceleration. Finite‐error performance would still need to be confirmed by forward electromagnetic simulations, and application to a specified process would require validating both the surrogate and the device under the relevant perturbations.

## 5 Conclusion

The neural adjoint method has been introduced as a neural‐operator‐based approach that targets a bottleneck in computational meta‐optics: the repeated full‐wave forward/adjoint simulations required for 3D adjoint optimization. Instead of predicting only scalar objectives, the method learns to directly infer dense 3D adjoint gradient fields on voxelized device domains, enabling rapid iterative refinement whilst preserving the physically meaningful update directions that drive topology optimization.

A stage‐wise Fourier neural operator has been proposed that performs coarse‐to‐fine residual refinement with progressively higher‐frequency emphasis, improving the recovery of localized high‐magnitude sensitivity peaks that strongly influence topology updates. To support rigorous analysis and reproducibility, a domain‐specific dataset has been constructed from conventional adjoint formulations using full‐wave FDTD simulations, pairing voxelized designs with solver‐generated volumetric adjoint gradients. Across three physically distinct inverse‐design benchmarks (RGB color router, achromatic metalens, and waveguide mode converter), surrogate‐driven optimization reaches solutions that remain close to FDTD adjoint optimization in final FoM whilst reducing design time from hours to seconds.

These results suggest a practical route toward interactive volumetric meta‐optics design, bridging recent advances in neural operators with the stringent fidelity requirements of photonic inverse design.

## Author Contributions

**Chanik Kang:** conceptualization, methodology, software, data curation, investigation, validation, formal analysis, visualization, writing – original draft, writing – review and editing, project administration. **Hyewon Suk:** investigation, data curation, visualization, writing – review and editing, software. **Ikbeom Jang:** writing – review and editing, supervision, project administration. **Haejun Chung:** resources, project administration, writing – review and editing, supervision, funding acquisition, visualization, conceptualization.

## Supporting information


Supporting Information S1

## Data Availability

The data that support the findings of this study are available from the corresponding author upon reasonable request.
