# Supplementary material for: Neural Adjoint Inverse Design of Three‐Dimensional Nanophotonic Devices
Source: Nanophotonics. 2026 Oct 16;15(20):e70334. doi: 10.1002/nap2.70334 (PMC13673514; doi:10.1002/nap2.70334)
Supplement: Supplementary file 1 [file NAP2-15-e70334-s001.pdf]

Supporting Information: Neural Adjoint Inverse Design of Three-Dimensional Nanophotonic Devices

Chanik Kang Hyewon Suk Ikbeom Jang Haejun Chung**

C. Kang
Department of Artificial Intelligence, Hanyang University, Seoul, Korea
H. Suk
Department of Artificial Intelligence Semiconductor Engineering, Hanyang University, Seoul, Korea
I. Jang
Department of Computer Engineering, Hankuk University of Foreign Studies, Yongin, Korea
Email Address: ijang@hufs.ac.kr
H. Chung
Department of Electronic Engineering, Hanyang University, Seoul, Korea
Department of Artificial Intelligence, Hanyang University, Seoul, Korea
Department of Artificial Intelligence Semiconductor Engineering, Hanyang University, Seoul, Korea
Email Address: haejun@hanyang.ac.kr

1 Additional Methods Details

1.1 Dataset Construction and Training Protocol

A domain-specific dataset is constructed consisting of paired permittivity volumes and adjoint sensitivity fields. Unlike generic PDE benchmarks that emphasize smooth state solutions [1, 2], the supervision used here consists of dense adjoint gradient fields, which exhibit sharp, localized sensitivity features induced by interference and resonant effects in meta-optics structures. Depending on the task and labeling cost, 600 waveguide, 1,100 metalens, and 3,300 color-router sample pairs are used for training and validation. For the color router, 5,000 volumes are generated in total, of which 3,000 are used for training and 300 for validation (Sec. 1.3). The waveguide (500/100) and metalens (1,000/100) splits use all generated samples. The final training sizes were selected with the dataset-size ablation of Sec. 5.1 (Table S3). The surrogate is intended to reproduce the FDTD adjoint gradient as closely as possible. For each task, the training set was therefore grown incrementally, with the architecture and training protocol held fixed. Along the measured range, the marginal gain in mean validation cosine similarity per added sample decreases monotonically, while each additional sample requires paired 3D forward/adjoint FDTD solves. The selected sizes are the points where the mean similarity enters the high-fidelity regime (82–88%) and further data yield diminishing gains, balancing fidelity against labeling and training cost. This fidelity level proved sufficient for surrogate-driven optimization to closely reproduce FDTD results (main text, Table 2). For each sample, the ground-truth adjoint gradient field is computed using the open-source FDTD package Meep [3] via paired forward and adjoint simulations. Fixed train/validation/test splits with a fixed random seed are used for reproducibility. All samples are represented on a fixed voxel grid and normalized to a consistent permittivity range, with voxels outside the design domain masked consistently across tasks; the target adjoint gradient labels are normalized using the mean and standard deviation calculated from the respective training datasets to stabilize the regression of high-dynamic-range gradient fields.

A unified training protocol and identical optimization hyperparameters are adopted across the three inverse-design domains. All models are implemented in PyTorch and trained on a single NVIDIA H200 GPU using the AdamW optimizer [4] with a cosine-annealing learning-rate schedule [5] and the Gaussian Error Linear Unit (GELU) activation across all stages [6]. The optimal checkpoint for each model is selected based on the minimum validation loss recorded on a held-out set of previously unseen samples. Complete hyperparameter values (epochs, batch size, learning rates, weight decay) are listed in Section 3 (Table S2).

1.2 Network Architectures

Four architectures are compared across the three inverse-design domains: a compact single-pass Fourier neural operator (FNO) baseline [1, 7]; a 3D U-Net with encoder–decoder skip connections (two residual blocks per level, GELU activation, Group Normalization, and strided convolutions in place of max-pooling); a Double-Stage FNO implementing a two-stage SW-FNO with a progressive Fourier-mode budget (low $\rightarrow$ high); and the proposed Triple-Stage FNO, which uses a three-stage cascade with coarse-to-fine mode budget (low $\rightarrow$ mid $\rightarrow$ high) for finer residual decomposition of boundary-aligned and resonant features. To enable a fair comparison of model capacity, the U-Net is sized so that its total parameter count approximately matches the Double-Stage FNO, isolating the contribution of multi-stage residual learning from that of simply scaling a monolithic single-pass architecture. Per-domain configurations (layer counts, channel widths, Fourier-mode budgets, and parameter counts) are listed in Section 2 (Table S1).

1.3 Simulation Setup

For the RGB color router, the design region is discretized on a voxel grid of size $28 \times 28 \times 20$, corresponding to a physical volume of $1.4\,\mu\text{m} \times 1.4\,\mu\text{m} \times 1.0\,\mu\text{m}$, with a simulation resolution of 20 pixels/μm. TiO_2 ($\epsilon = 7.0$) is used as the design-region material embedded within an SiO_2 ($\epsilon = 2.1$) background, with Si photodiode pixels ($\epsilon = 11.8$) arranged in a Bayer pattern [8, 9]. To ensure structural diversity, 5,000 paired samples are generated, with the permittivity volumes initialized by Gaussian filtering of random noise (filter width σ randomly sampled from $[1.0, 4.0]$). The adjoint gradient fields are computed for three wavelengths corresponding to the RGB channels ($\lambda = 450, 550, 650\,\text{nm}$) and summed to form the multi-objective supervision signal; 3,000 samples are used for training and 300 for validation.
For the achromatic metalens, the design region is discretized on a voxel grid of size $100 \times 100 \times 10$, corresponding to a physical volume of $5.0\,\mu\text{m} \times 5.0\,\mu\text{m} \times 0.5\,\mu\text{m}$. The simulation resolution is 20 pixels/μm, with TiO_2 ($\epsilon = 7.0$) as the high-index metasurface material on an SiO_2 ($\epsilon = 2.1$) substrate. The objective maximizes the field intensity at a focal point located $3.0\,\mu$m away from the design region across the visible spectrum ($\lambda = 450, 550, 650\,\text{nm}$). As for the colour router, the adjoint gradient fields are computed at the three target wavelengths and summed to form the multi-objective supervision signal. 1,100 paired samples are generated using Meep, with the permittivity volumes initialized using a larger filter width $\sigma \in [2.0, 6.0]$ to account for the larger spatial scale; 1,000 samples are used for training and 100 for validation.
For the waveguide mode converter, a 3D silicon-on-insulator slab structure is used [10], with the design region discretized on a voxel grid of size $80 \times 30 \times 5$, corresponding to a physical volume of $4.0\,\mu\text{m} \times 1.5\,\mu\text{m} \times 0.25\,\mu\text{m}$. Silicon ($n = 3.48$) is used for the waveguide core and silica ($n = 1.44$) for the cladding. The optimization objective maximizes the conversion efficiency from the fundamental TE_0 mode to the first-order TE_1 mode at a telecommunication wavelength of $\lambda = 1.55\,\mu$m. The gradient is computed at this single wavelength, corresponding to the $K = 1$ case of the multi-wavelength formulation. 600 paired samples are generated using Meep. Owing to the elongated geometry along the propagation axis (x-direction) and the thin vertical cross-section (z-direction), the Fourier mode budget in the SW-FNO is configured with anisotropic allocation: higher mode counts along the longitudinal and transverse axes to capture complex mode-coupling interference, and a constrained vertical-axis budget reflecting the localized slab physics; 500 samples are used for training and 100 for validation.

2 Network Architecture Specifications

This section reports the complete architectural configurations of all four models evaluated in the main text: the compact FNO, the 3D U-Net, the Double-Stage FNO, and the proposed Triple-Stage FNO (SW-FNO). Configurations are reported per domain to reflect the differing voxel geometries of the color router ($28 \times 28 \times 20$), the metalens ($100 \times 100 \times 10$), and the waveguide ($80 \times 30 \times 5$). Table S1 summarizes the number of stages, layer counts, channel widths, Fourier-mode budgets along each spatial axis, and

Table S1: Architectural configurations of all compared models across the three inverse-design domains. For the multi-stage models, the listed modes are per-stage cutoffs. Each stage retains all Fourier modes up to its own cutoff, and the low/mid/high emphasis arises from residual training rather than from an architectural band restriction.

Domain		FNO	U-Net	Double-Stage FNO	Triple-Stage FNO
Color Router	#Stages	1	–	2 (L→H)	3 (L→M→H)
	Layers	4	4	4	4
	Channels	32	init: 32	32	32
	Fourier modes	[10,10,8]	–	S1:[6,6,6]/S2:[12,12,10]	S1:[6,6,6]/S2:[10,10,8]/S3:[12,12,10]
	#Params (M)	2.06	4.10	4.16	6.22
Metalens	#Stages	1	–	2 (L→H)	3 (L→M→H)
	Layers	4	5	4	4
	Channels	32	init: 60	32	32
	Fourier modes	[24,24,4]	–	S1:[16,16,3]/S2:[32,32,4]	S1:[16,16,3]/S2:[24,24,4]/S3:[32,32,4]
	#Params (M)	7.09	14.71	14.71	21.80
Waveguide	#Stages	1	–	2 (L→H)	3 (L→M→H)
	Layers	4	3	4	4
	Channels	32	init: 40	32	32
	Fourier modes	[40,9,2]	–	S1:[24,7,2]/S2:[52,11,2]	S1:[24,7,2]/S2:[40,9,2]/S3:[52,11,2]
	#Params (M)	2.96	6.15	6.09	9.05

total parameter counts. The Fourier-mode budget for the waveguide is configured with anisotropic allocation: high mode counts along the longitudinal (x) and transverse (y) axes to capture complex mode-coupling interference, with the vertical (z) budget constrained to reflect the localized slab physics.

2.1 Selection of the Stage-Wise Mode Cutoffs

The per-stage cutoffs in Table S1 follow the rule stated in Sec. 3.1 of the main text. The mid-stage cutoff coincides with the single-stage FNO baseline in every task ([10,10,8] for the color router, [24,24,4] for the metalens, and [40,9,2] for the waveguide), so the cascade is anchored to a capacity known to perform well in a single pass. The Stage-1 cutoff retains roughly 60–78% of the baseline cutoff per axis (S1 $\approx$ 0.60–0.78 $\times$ S2), which restricts the first stage to the coarse global scattering envelope. The Stage-3 cutoff extends 20–33% beyond the baseline (S3 $\approx$ 1.2–1.33 $\times$ S2), approaching the largest mode count representable on the voxel grid, and axes already at their grid limit are kept fixed across stages, e.g., $k_z = 2$ for the waveguide. The Double-Stage variant uses the Stage-1 and Stage-3 cutoffs and removes the intermediate refinement stage, serving as the stage-granularity control for the stage ablation in the main text (Table 3). The specific ratios are heuristic rather than derived from first principles, and their adequacy is established empirically by the same ablation.

3 Training Hyperparameters

All models are implemented in PyTorch and trained on a single NVIDIA H200 GPU. The same training protocol is used for all five architectures across all three domains.

4 Scope and Limitations

The training data are generated under a fixed simulation configuration (resolution, materials, boundary conditions) and from a controlled distribution of synthetic structures. Consequently, the learned surrogate reflects the assumptions and coverage of this training domain. Three points clarify the scope of the present work and the boundary between this contribution and adjacent research directions.

Table S2: Training hyperparameters used for all models across the three inverse-design tasks.

Hyperparameter	Value
Optimizer	AdamW
Epochs	300
Batch size	8
Initial learning rate	8×10^{-3}
AdamW coefficients	$(\beta_1, \beta_2) = (0.9, 0.999)$
AdamW ϵ	10^{-8}
Weight decay	10^{-4}
Learning-rate schedule	Cosine annealing
Minimum learning rate	10^{-5}
Activation	GELU
Loss	Mean-squared error on normalized gradient labels
Checkpoint selection	Minimum validation loss on held-out set
Hardware	NVIDIA H200 GPU (single device)
Framework	PyTorch

4.1 Orthogonality with Robust Topology Optimization

The focus of the present work is on accelerating gradient evaluation, not on prescribing the objective function. The surrogate predicts the dense per-voxel adjoint gradient $\nabla_{\mathbf{x}}\mathcal{J}$ regardless of whether $\mathcal{J}$ corresponds to a vanilla figure-of-merit or a fabrication-aware/robust formulation. Robust topology optimization techniques such as density filtering, projection schemes, three-field formulations, and length-scale control modify the objective itself rather than the gradient evaluation routine. Because the proposed surrogate operates on the gradient evaluation, these formulations compose orthogonally: any robust formulation yields a gradient with the same dense voxel-wise structure, which the surrogate can be trained to predict without architectural changes. The design updates in this work use threshold projection in its standard optimization role (Sec. 6); a density filter is available in the same pipeline but was not applied in the reported runs. Imposing fabrication-driven settings on the same machinery, such as a filter radius chosen from a minimum feature size or three-field robust formulations, is a direct extension and is left for future work. Because surrogate inference takes milliseconds, the additional gradient evaluations required by such formulations, or by perturbation-based tolerance screening of a finished design, would be computationally inexpensive. Freeform volumetric designs retain a complementary role in this workflow. They estimate the performance ceiling of a given design volume and material system, against which fabrication-constrained designs can be benchmarked.

4.2 Out-of-Distribution Generalization

Out-of-distribution (OOD) generalization across substantially different materials, wavelengths, or domain sizes is an open question for any operator-learning approach and is not the primary focus of this study. For the wavelength axis in particular, wavelength-conditioned surrogates, with wavelength as an additional input channel or with per-wavelength output heads, are a natural extension for tunable spectral objectives and require no change to the stage-wise architecture. The mesh-agnostic property of FNO suggests promising directions for cross-scale generalization, in which a surrogate is trained on smaller, simulation-feasible subdomains and then applied to predict gradients on much larger volumes. Such an extension lies outside the present scope and is left for future investigation.

4.3 Solver-Reduced versus Solver-Free Operation

The present evaluation is restricted to relatively compact volumetric domains. Although neural adjoint inference can substantially reduce reliance on full-wave simulations during iterative design, the current pipeline is best described as solver-reduced rather than fully solver-free, since solver-generated labels are required for training. A natural direction for future work is to leverage the scalability of neural operators

to enable transfer from small training subdomains to much larger design volumes, which could further decouple optimization throughput from the cost of full-wave simulation.

5 Additional Ablation Studies

5.1 Dataset-Size Dependence

To examine how performance scales with the number of training examples, the proposed Triple-Stage FNO is retrained on subsets of the original training pool for each of the three inverse-design tasks, while keeping the network architecture, optimizer, learning-rate schedule, and validation protocol fixed. Cosine similarity between predicted and ground-truth adjoint gradient fields on the held-out validation set is reported in Table S3. Both the mean (with standard deviation in parentheses) and the maximum across the validation set are reported, since the mean captures the typical quality of predicted gradients used by the optimizer, whereas the maximum reflects the best-case alignment achievable on individual samples.

Table S3: Dataset-size dependence of the proposed Triple-Stage FNO. Values are cosine similarity (%) between predicted and ground-truth adjoint gradient fields on the validation set, reported as mean (standard deviation) and maximum. The architecture and hyperparameters are kept fixed across training-set sizes.

	Color Router				Metalens				Waveguide			
# Training samples	500	1,000	3,000	4,000	100	300	500	1,000	100	300	400	500
Mean (Std)	26.0 (34.2)	43.9 (38.0)	82.3 (13.7)	85.2 (9.8)	2.3 (14.7)	36.5 (26.8)	57.0 (28.0)	87.6 (7.5)	21.1 (39.2)	57.9 (46.5)	76.2 (40.0)	87.9 (30.3)
Max	99.3	98.0	99.8	99.3	45.8	93.5	97.7	98.2	98.9	99.2	99.7	99.4

Three observations emerge from Table S3. First, all three tasks exhibit strong data-size dependence in the mean cosine similarity: 26.0% to 85.2% as the color-router training set grows from 500 to 4,000 samples, 2.3% to 87.6% across the metalens range (100 to 1,000 samples), and 21.1% to 87.9% across the waveguide range (100 to 500 samples). Second, the standard deviation of the cosine similarity decreases as more training data are added (e.g., 34.2 to 13.7 for the color router and 14.7 to 7.5 for the metalens), indicating that additional data improves not only the average gradient fidelity but also the consistency of the surrogate across diverse devices, which is the property most relevant for reliable gradient-based optimization. Third, the maximum cosine similarity saturates much earlier than the mean: best-case alignments above 98% are already achieved at the smallest training sizes for the color router and waveguide, indicating that the model can fit individual gradient fields with limited supervision, but a sufficiently large and diverse training set is required to make this fidelity hold across the validation distribution. The metalens task, which has the largest design region and therefore the highest sensitivity-field dimensionality, requires the largest training set to reach the same regime: the mean similarity exceeds 85% only at 1,000 samples, consistent with the higher representational demand of broadband focusing in a thin slab. The 4,000-sample color-router entry further shows that gains diminish beyond the selected size. Adding 1,000 samples raises the mean similarity by only 2.9 percentage points (82.3% to 85.2%), roughly an order of magnitude less per sample than the preceding interval, while the standard deviation continues to contract (13.7 to 9.8). The main results therefore retain 3,000 training samples, balancing fidelity against labeling cost.

6 Design-Update Rule and Shared Optimization Hyperparameters

The surrogate-driven optimization and the FDTD-adjoint reference share an identical design-update rule. The only difference between the two pipelines is the source of the gradient field: paired forward/adjoint FDTD solves for the reference, and a single surrogate inference for the Neural Adjoint Method.
Both pipelines maintain a latent (unconstrained) design variable ρ and use the standard threshold-projection parameterization of topology optimization [11, 12, 13]. At every iteration, the latent field is mapped to

the physical design by a smoothed-threshold projection,

$$\bar{\rho} = \frac{\tanh(\beta\eta) + \tanh\big(\beta(\rho - \eta)\big)}{\tanh(\beta\eta) + \tanh\big(\beta(1 - \eta)\big)}, \tag{S1}$$

which drives the design toward binary permittivity values. The projection sharpness follows a linear continuation from $\beta = 1$ to $\beta = 32$ over the run, with $\eta = 0.5$. A density filter for minimum-feature-size control is available in the same pipeline but was not applied in the reported runs, so the parameterization reduces to the projection alone. The gradient with respect to the physical design, $\mathbf{g} = \partial\mathcal{J}/\partial\bar{\rho}$, is supplied either by the FDTD adjoint solve or by the surrogate, after inverting the dataset-level label normalization of Sec. 1.1. It is backpropagated through the projection by the chain rule, and the latent variable is updated by a gradient-based optimizer,

$$\nabla_\rho \mathcal{J} = \left(\frac{\partial\bar{\rho}}{\partial\rho}\right)^{\top} \mathbf{g}, \qquad \rho \leftarrow \mathrm{Opt}(\rho,\, \nabla_\rho\mathcal{J};\, \alpha)\,, \tag{S2}$$

for a fixed number of iterations (50 for the color router, 100 for the metalens, and 30 for the waveguide; main text, Table 2), starting from an identical random initial permittivity volume for both pipelines. The optimizer, step size α, and projection parameters are identical for both pipelines and are listed in Table S4. In implementation, the chain rule of Eq. (S2) is realized by backpropagating the pseudo-objective $-\sum \hat{\mathbf{g}} \odot \bar{\rho}$ with the predicted gradient treated as a constant, which yields exactly the stated update. Because this chain rule is applied by automatic differentiation outside the network, the surrogate is queried in the same form it was trained on, namely the physical (projected) design, and does not need to represent the parameterization itself.

Table S4: Design-update hyperparameters, identical for the surrogate-driven and FDTD-adjoint pipelines.

Parameter	Value
Optimizer (design updates)	Adam ($\beta_1 = 0.9$, $\beta_2 = 0.999$)
Step size α	0.01 (fixed; no schedule)
Projection β	linear continuation, 1.0 $\to$ 32.0
Projection η	0.5
Initialization	identical random permittivity volume for both pipelines

6.1 Gradient-Magnitude Fidelity

The cosine similarity reported in the main text measures directional fidelity. To quantify magnitude fidelity separately, the norm ratio $\|\hat{\mathbf{g}}\|/\|\mathbf{g}\|$ is evaluated over the color-router validation set (300 samples), where $\hat{\mathbf{g}}$ is the surrogate prediction after inverse normalization and $\mathbf{g}$ the FDTD adjoint gradient. The distribution is shown in Fig. S1 and summarized in Table S5. The ratio is centred at 0.86 (median) with an interquartile range of 0.81–0.91 and a 5th–95th percentile range of 0.73–0.95; 4 of 300 samples exceed 1.0 (maximum 1.80). The surrogate thus mildly and consistently underestimates the overall gradient magnitude, a typical shrinkage of mean-squared-error regression. Because the deviation is close to a global scale factor, it is absorbed by the optimizer step size and does not alter the update direction, consistent with the directional metric adopted in the main text. This analysis reuses stored validation predictions and requires no additional full-wave simulations.

Table S5: Gradient-magnitude fidelity $\|\hat{\mathbf{g}}\|/\|\mathbf{g}\|$ on the color-router validation set.

Task	Samples	Median	IQR	P5–P95	Mean (Std)
Color router	300	0.86	0.81–0.91	0.73–0.95	0.86 (0.09)

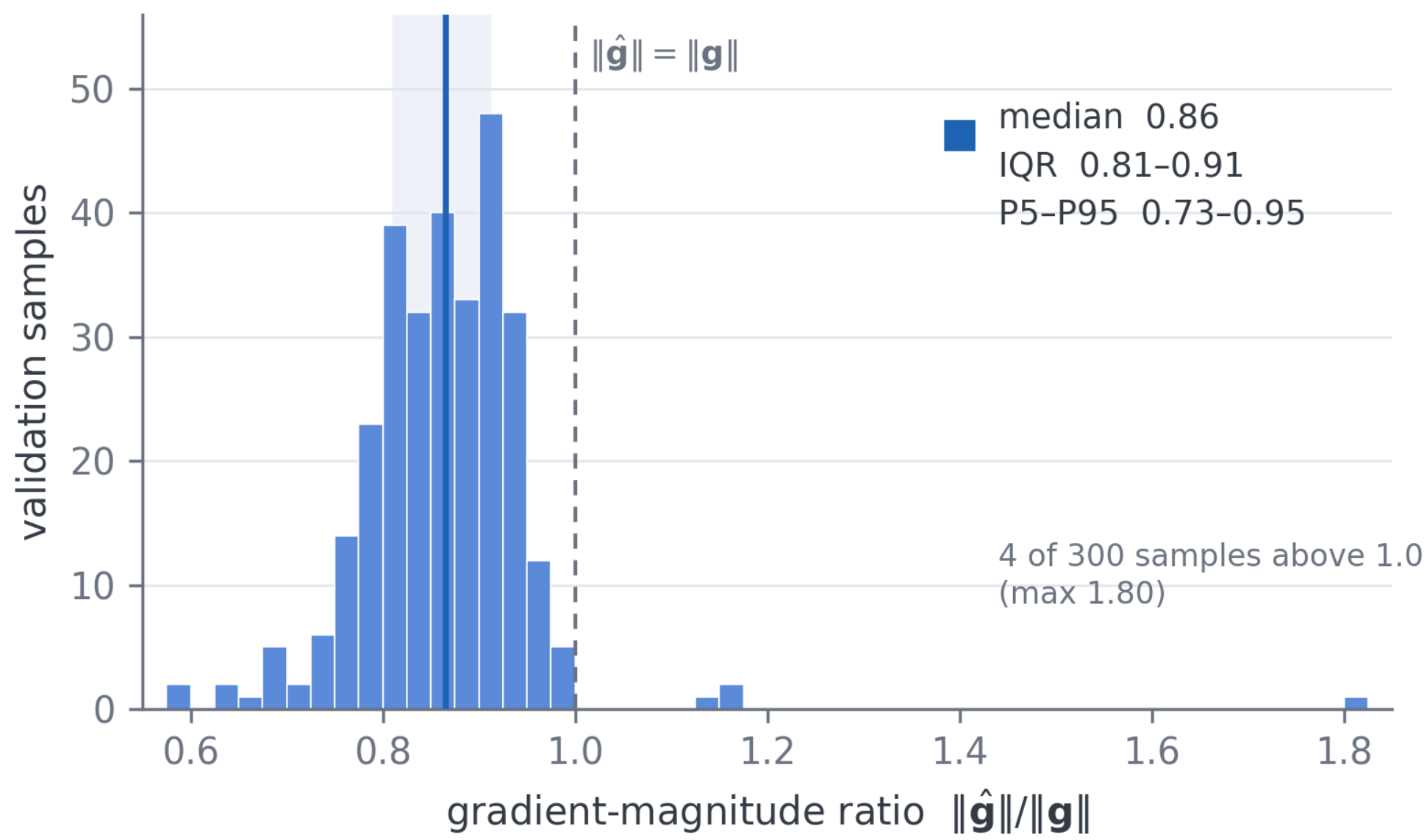

Figure S1: Distribution of the gradient-magnitude ratio $\|\hat{\mathbf{g}}\|/\|\mathbf{g}\|$ over the color-router validation set (300 samples). The solid line marks the median (0.86), the shaded band the interquartile range (0.81–0.91), and the dashed line exact magnitude reproduction.

7 Coverage of Optimization Trajectories

The surrogate is deployed along optimization trajectories, so the relevant question is how well the synthetic training distribution covers the structures that optimization actually visits. This section assesses this coverage for the representative color-router task with a spectral analysis of a surrogate-driven optimization run under the identical protocol of Table S4; the analysis requires no additional full-wave simulations. Whether the observed late-phase mismatch affects optimization fidelity is then assessed through the end-to-end trajectory comparison already reported in the main text (Fig. 5a, Table 2).
The synthetic training distribution is constructed with this deployment in mind. Training volumes are generated by Gaussian filtering of random noise with a per-sample filter width $\sigma \in [1.0, 4.0]$ voxels (Sec. 1.3), producing an ensemble that spans coarse to fine structures. This matches the coarse-to-fine character of topology evolution during optimization (main text, Fig. 5c): early iterates are smooth and dominated by low spatial frequencies, whereas later iterates progressively develop fine, high-spatial-frequency features. The optimization is initialized from a volume of the same Gaussian-filtered family, so trajectories start in-distribution by construction, and whether the subsequent iterates remain within the training envelope is assessed below.

7.1 Spectral coverage of trajectory iterates

Trajectory iterates are compared against the training ensemble (500 samples) in two measures: the radially averaged spatial power spectral density and the correlation length. The initial iterate lies within the 5–95% training envelope in both measures, with a correlation length of 3.7 voxels against a training range of 1.7–4.9 voxels. Iterates remain within or near the envelope for roughly the first half of the run, and the correlation length leaves the training range around iteration 21. In the second half, the projection drives the design toward binary values, and the iterates develop fine, near-binary features whose high-spatial-frequency content lies above the smooth synthetic envelope, with the correlation length reaching 0.8 voxels at iteration 50. The synthetic distribution therefore covers the structure-forming phase of the trajectory well, while the late, strongly binarized iterates progressively exceed it. This mismatch is intrinsic to any smooth synthetic ensemble paired with binarized designs; whether it affects optimization fidelity is assessed next.

7.2 End-to-end trajectory validation

Precisely because the late iterates leave the training envelope, the decisive question is whether optimization fidelity survives that phase. The surrogate-driven run and the FDTD-adjoint reference start from an identical initialization and share the identical update rule (Table S4), and their FoM trajectories remain closely matched over all 50 iterations, including the binarization phase in which the iterates leave the training envelope; the surrogate-driven run attains 95.9% of the FDTD-optimized FoM (main text, Table 2). A systematic failure of the surrogate gradients on late, near-binary structures would appear as stagnation or divergence of the surrogate-driven FoM trajectory in precisely that phase, which is not observed. The late-phase spectral mismatch therefore does not translate into a loss of optimization fidelity.

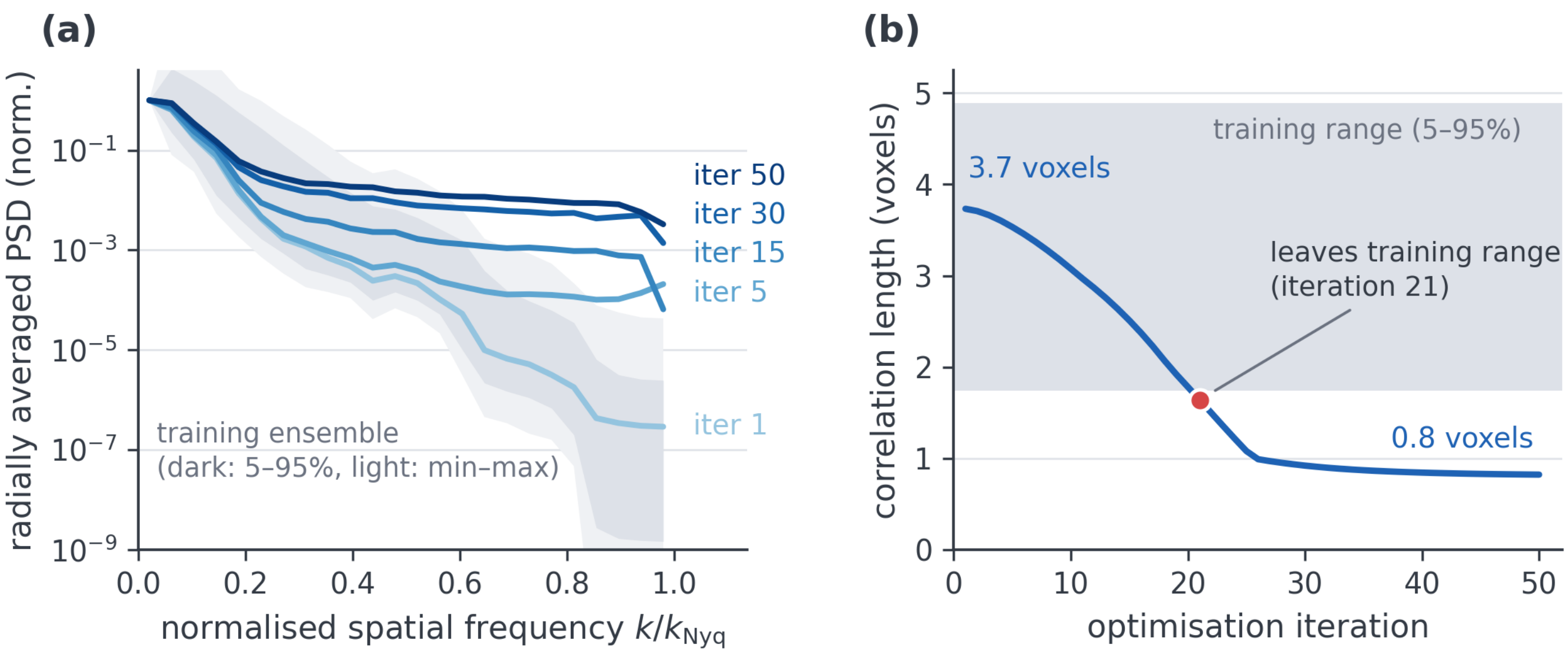

Figure S2: Spectral coverage of trajectory iterates (color router). (a) Radially averaged spatial power spectral density of iterates at several optimization stages (blue, light to dark) against the training-ensemble envelope (gray: 5–95% and min–max ranges over 500 training samples); profiles are normalized to their lowest-frequency bin. (b) Correlation length of the iterates versus the 5–95% training range (gray band); the trajectory starts inside the range (3.7 voxels), leaves it around iteration 21 as binarization proceeds, and reaches 0.8 voxels at iteration 50.

References

[1] Z. Li, N. Kovachki, K. Azizzadenesheli, et al., Fourier neural operator for parametric partial differential equations, *arXiv preprint arXiv:2010.08895* **2020**.

[2] M. Takamoto, T. Praditia, R. Leiteritz, et al., Pdebench: An extensive benchmark for scientific machine learning, *Advances in neural information processing systems* **2022**, *35* 1596–1611.

[3] A. F. Oskooi, D. Roundy, M. Ibanescu, P. Bermel, J. Joannopoulos, S. G. Johnson, Meep: A flexible free-software package for electromagnetic simulations by the FDTD method, *Computer Physics Communications* **2010**, *181*, 3 687–702.

[4] I. Loshchilov, F. Hutter, Decoupled weight decay regularization, *arXiv preprint arXiv:1711.05101* **2017**.

[5] I. Loshchilov, F. Hutter, Sgdr: Stochastic gradient descent with warm restarts, *arXiv preprint arXiv:1608.03983* **2016**.

[6] D. Hendrycks, K. Gimpel, Gaussian error linear units (gelus), *arXiv preprint arXiv:1606.08415* **2016**.

[7] V. Duruisseaux, J. Kossaifi, A. Anandkumar, Fourier Neural Operators Explained: A Practical Perspective, *arXiv preprint arXiv:2512.01421* **2025**.

[8] B. Bayer, Color imaging array, *United States Patent, no. 3971065* **1976**.

[9] S. Lee, J. Hong, J. Kang, et al., Inverse design of color routers in CMOS image sensors: toward minimizing interpixel crosstalk, *Nanophotonics* **2024**, *13*, 20 3895–3914.

[10] A. Y. Piggott, J. Lu, K. G. Lagoudakis, J. Petykiewicz, T. M. Babinec, J. Vučković, Inverse design and demonstration of a compact and broadband on-chip wavelength demultiplexer, *Nature photonics* **2015**, *9*, 6 374–377.

[11] F. Wang, B. S. Lazarov, O. Sigmund, On projection methods, convergence and robust formulations in topology optimization, *Structural and multidisciplinary optimization* **2011**, *43*, 6 767–784.

[12] R. E. Christiansen, O. Sigmund, Inverse design in photonics by topology optimization: tutorial, *Journal of the Optical Society of America B* **2021**, *38*, 2 496–509.

[13] A. M. Hammond, A. Oskooi, S. G. Johnson, S. E. Ralph, Photonic topology optimization with semiconductor-foundry design-rule constraints, *Optics Express* **2021**, *29*, 15 23916–23938.
